# Association of the *OCA2* Polymorphism His615Arg with Melanin Content in East Asian Populations: Further Evidence of Convergent Evolution of Skin Pigmentation

**DOI:** 10.1371/journal.pgen.1000867

**Published:** 2010-03-05

**Authors:** Melissa Edwards, Abigail Bigham, Jinze Tan, Shilin Li, Agnes Gozdzik, Kendra Ross, Li Jin, Esteban J. Parra

**Affiliations:** 1 Department of Anthropology, University of Toronto at Mississauga, Mississauga, Ontario, Canada; 2 Department of Pediatrics, University of Washington, Seattle, Washington, United States of America; 3 School of Life Sciences, Fudan University, Shanghai, China; University of Oxford, United Kingdom

## Abstract

The last decade has witnessed important advances in our understanding of the genetics of pigmentation in European populations, but very little is known about the genes involved in skin pigmentation variation in East Asian populations. Here, we present the results of a study evaluating the association of 10 Single Nucleotide Polymorphisms (SNPs) located within 5 pigmentation candidate genes (*OCA2*, *DCT*, *ADAM17*, *ADAMTS20*, and *TYRP1*) with skin pigmentation measured quantitatively in a sample of individuals of East Asian ancestry living in Canada. We show that the non-synonymous polymorphism rs1800414 (His615Arg) located within the *OCA2* gene is significantly associated with skin pigmentation in this sample. We replicated this result in an independent sample of Chinese individuals of Han ancestry. This polymorphism is characterized by a derived allele that is present at a high frequency in East Asian populations, but is absent in other population groups. In both samples, individuals with the derived G allele, which codes for the amino acid arginine, show lower melanin levels than those with the ancestral A allele, which codes for the amino acid histidine. An analysis of this non-synonymous polymorphism using several programs to predict potential functional effects provides additional support for the role of this SNP in skin pigmentation variation in East Asian populations. Our results are consistent with previous research indicating that evolution to lightly-pigmented skin occurred, at least in part, independently in Europe and East Asia.

## Author Summary

Our knowledge of the genetic basis of normal pigmentation variation in human populations is quite incomplete. Recent studies have identified some of the genes responsible for the reduction in melanin content in European populations, but this is not the case for other population groups, such as East Asians. Here, we report that a genetic variant located within the gene *OCA2* (rs1800414) is associated with skin pigmentation in two samples of East Asian ancestry. The allele associated with lower melanin levels is found at high frequencies in East Asian populations, but is absent or at very low frequencies in other population groups. This is one of the first reports of association of genetic markers with quantitative measures of pigmentation in East Asian populations and it confirms previous evidence indicating that evolution towards light skin occurred, at least in part, independently in Europe and East Asia. The *OCA2* gene has been under positive selection in Europe and East Asia, but different alleles have been selected in each region.

## Introduction

The remarkable variation observed in skin, hair and iris pigmentation in human populations is the result of differences in the amount, type and distribution of the pigment melanin, which is synthesized by specialized cells known as melanocytes. Pigmentation is a complex trait, influenced by numerous genes and their interactions. The last decade has witnessed impressive advances in our understanding of the genetics of normal pigmentation variation, driven by functional studies, gene expression studies, studies in animal models, analyses of signatures of natural selection and candidate gene or genome-wide association studies. At least 11 genes are known to be associated with normal pigmentation variation: *TYR*, *TYRP1*, *OCA2/HERC2*, *SLC45A2*, *SLC24A5*, *SLC24A4*, *MC1R*, *ASIP*, *KITLG*, *IRF4* and *TPCN2* [Reviewed in 1],[2]. In European and related populations, a clear picture is emerging of the genetic and evolutionary processes associated with skin lightening. The most important genes involved are *SLC24A5*, *SLC45A2* and *KITLG*, which explain a large portion of the skin pigmentation differences observed between European and West African populations [3]–[6]. Other polymorphisms within the genes *TYR*, *OCA2*, *MC1R*, *ASIP* and *IRF4* are known to play a role in normal pigmentation variation in populations of European descent [7]–[13]. A recent genome-wide study reported that variants within the genes *SLC24A5*, *SLC45A2* and *TYR* are associated with skin pigmentation in a South Asian sample [14]. Interestingly, the genes *SLC24A5* and *SLC45A2* show an extremely unusual pattern of allele frequency distribution, with a derived allele near fixation in European populations, and the alternative ancestral allele fixed in other population groups [5]. These two genes show strong signatures of selection in European populations [15]–[19], but not in other population groups. These observations indicate that evolution to lightly-pigmented skin happened, at least in part, independently in Europe and East Asia [5],[16]. Unfortunately, while there have been important advances in our understanding of the genetics of pigmentation in European populations, very little is known about the genes involved in skin pigmentation variation in East Asian populations. There are also pigmentation candidate genes (*DCT*, *ADAM17*, *ADAMTS20* , *KITLG*, *TYRP1* and *OCA2*) that show signatures of selection in East Asians [16]–[21], but formal association studies are required to confirm that these genes are involved in skin pigmentation variation. Studies of signatures of natural selection are extremely useful as a strategy to identify potential genes of interest, but it is critical to carry out further analysis in order to confirm that the signatures of selection are not false positives and to eliminate the possibility that positive selection was related to biological processes other than pigmentation [22],[23]. For example, the *ADAM17* gene has been implicated in many processes involved in cell-cell and cell-matrix interactions, including fertilization, muscle development and neurogenesis. Therefore, it is in principle possible that the signatures of selection observed in this gene are due to its role in these processes.

Here, we present the results of a study evaluating the association of 10 Single Nucleotide Polymorphisms (SNPs) located within 5 pigmentation candidate genes (*OCA2*, *DCT*, *ADAM17*, *ADAMTS20* and *TYRP1*) with skin pigmentation measured quantitatively in a sample of individuals of East Asian ancestry living in Canada. We selected these loci based on previous studies that identified signatures of natural selection in East Asian populations, and prioritized a list of SNPs within these genes using the program SNPSelector. We show that the non-synonymous polymorphism rs1800414 located within the *OCA2* gene is significantly associated with skin pigmentation in this East Asian sample. We replicated this result in an independent sample of Chinese individuals of Han ancestry. This polymorphism is characterized by a derived allele that is present at high frequency in East Asian populations, but is absent in other populations. In our sample, individuals with the derived G allele, which codes for the amino acid arginine, show lower melanin levels than those with the ancestral A allele, which codes for the amino acid histidine. An analysis of this non-synonymous polymorphism using several programs to predict potential functional effects (see materials and methods) provides additional support for the role of this SNP in skin pigmentation variation in East Asian populations.

## Results

We applied four tests of positive selection based on different statistics to the five genes analyzed in this study. For these tests, we used genomewide information available for the HapMap East Asian, European and African samples (see Material and methods section). Table 1 shows the results of the four tests of natural selection in the HapMap sample. In accordance to previous reports [16]–[21], we observed evidence of positive selection in East Asian populations for these pigmentation genes. The *OCA2* gene shows numerous SNPs displaying high levels of differentiation in the East Asian sample with respect to the genomewide average (LSBL tests), very negative Tajima's *D* values for two windows encompassing a portion of this gene and a reduction in genetic diversity. We also observed clusters of markers exhibiting high differentiation for the *DCT* gene, as well as evidence of a reduction of genetic diversity in the East Asian sample for this locus (ln*RH* test). The *ADAM17* gene is significant for the LSBL, ln*RH* and Tajima's *D* tests, and is also significant for the WGLRH test, indicating that *ADAM17* has haplotypes characterized by derived alleles that have risen to very high frequencies and have longer than expected levels of Linkage Disequilibrium (LD). The gene *ADAMTS20* has extreme values for the LSBL and Tajima's *D* statistics. Finally, markers in the gene *TYRP1* show high levels of genetic differentiation between East Asians and the other two HapMap populations measured by LSBL and this locus is encompassed by a significant extended haplotype region (WGLRH test).

**Table 1:** Tests of positive selection for the five pigmentation genes analyzed in this study in the East Asian HapMap sample.

Gene	LSBL: Number of significant markers (p<0.001, p<0.01, p<0.05)	lnRH: Number of significant windows (p<0,001, p<0.01, p<0.05)	Tajima's D: Number of significant windows (p<0,001, p<0.01, p<0.05)	WGLRH p-value
*OCA2*	(8, 9, 16)	EAS/EUR (0, 0, 0)	(0, 2, 0)	NS
		EAS/WAF (0, 0, 1)		
*DCT*	(3, 2, 6)	EAS/EUR (0, 2, 3)	(0, 0, 0)	NS
		EAS/WAF (0, 0, 2)		
*ADAM17*	(5, 8, 4)	EAS/EUR (0, 0, 0)	(1, 3, 1)	p = 0.04
		EAS/WAF (0, 0, 1)		
*ADAMTS20*	(0, 1, 7)	EAS/EUR (0, 0, 0)	(0, 0, 2)	NS
		EAS/WAF (0, 0, 0)		
*TYRP1*	(0, 5, 6)	EAS/EUR (0, 0, 0)	(0, 0, 0)	p = 0.01
		EAS/WAF (0, 0, 0)		

Ten polymorphisms located within these five genes were genotyped in a sample of individuals of East Asian ancestry (N = 122). Table 2 reports the genotype and allele frequencies for each marker. No significant deviations from Hardy-Weinberg proportions were identified for any of the SNPs. We evaluated the patterns of LD between the markers located in each gene using an Expectation Maximization algorithm implemented in the program EMLD. LD was low between the markers located within the *OCA2* gene (rs7495174/rs1800414: r^2^ = 0.06; rs7495174/rs1545397: r^2^ = 0.05 and rs1800414/rs1545397: r^2^ = 0.25). In contrast, there was perfect LD between the markers located within the *DCT* gene (rs1407995/rs2031526: r^2^ = 1). Finally, within the *ADAMTS20* gene, there was almost perfect LD between the markers rs11182091 and rs11182085 (r^2^>0.99), but LD was substantially lower between rs11182091 and rs1510523 (r^2^ = 0.30) and rs11182085 and rs1510523 (r^2^ = 0.31).

**Table 2:** Observed and expected genotype frequencies, allele frequencies, and the Hardy-Weinberg exact test for 10 SNPs in the Canadian East Asian sample.

Gene	Polymorphism	Observed and Expected Genotype Frequencies	Allele Frequencies	Hardy-Weinberg Exact Test (p)
		A:A = 15 (15.64)	A = 0.360	
*OCA2*	rs7495174 (A/G)	A:G = 57 (55.72)	G = 0.640	1.000
		G:G = 49 (49.64)		
		A:A = 18 (20.01)	A = 0.408	
*OCA2*	rs1800414 (A/G)	A:G = 62 (57.98)	G = 0.592	0.571
		G:G = 40 (42.01)		
		A:A = 4 (3.28)	A = 0.164	
*OCA2*	rs1545397 (A/T)	A:T = 32 (33.44)	T = 0.836	0.739
		T:T = 86 (85.28)		
		C:C = 14 (10.42)	C = 0.293	
*DCT*	rs1407995 (C/T)	C:T = 43 (50.17)	T = 0.707	0.125
		T:T = 64 (60.42)		
		A:A = 63 (59.71)	A = 0.702	
*DCT*	rs2031526 (A/G)	A:G = 44 (50.58)	G = 0.298	0.190
		G:G = 14 (10.71)		
		C:C = 3 (2.39)	C = 0.140	
*ADAM17*	rs4328603 (C/T)	C:T = 28 (29.22)	T = 0.860	0.702
		T:T = 90 (89.39)		
		C:C = 34 (34.13)	C = 0.533	
*ADAMTS20*	rs11182091 (C/T)	C:T = 60 (59.73)	T = 0.467	1.000
		T:T = 26 (26.13)		
		C:C = 5 (8.13)	C = 0.258	
*ADAMTS20*	rs1510523 (C/T)	C:T = 53 (46.73)	T = 0.742	0.234
		T:T = 64 (67.13)		
		A:A = 36 (35.46)	A = 0.541	
*ADAMTS20*	rs11182085 (A/G)	A:G = 59 (60.09)	G = 0.459	0.856
		G:G = 26 (25.46)		
		A:A = 32 (29.01)	A = 0.492	
*TYRP1*	rs2075509 (A/C)	A:C = 54 (59.98)	C = 0.508	0.277
		C:C = 34 (31.01)		

**Figure 1:**
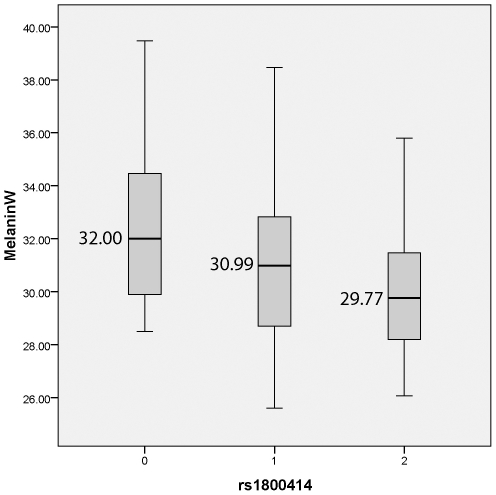
Boxplot showing melanin index by rs1800414 genotype for the Canadian East Asian sample. The top of the box is the 75^th^ percentile, the bottom of the box is the 25^th^ percentile and the line in the centre is the median. The lines extending from the box mark the highest and lowest melanin index measurements.

We tested if there was evidence of association between the 10 SNPs and quantitative measures of constitutive pigmentation (melanin index) in the East Asian sample. The results of the linear regression analysis for each marker, including sex as a covariate are depicted in Table 3. The rs1800414 polymorphism located within the *OCA2* gene showed a significant association with skin pigmentation. Using an additive model, we estimated that each copy of the G allele decreases skin pigmentation by approximately 1.3 melanin units (p = 0.002) and the rs1800414 polymorphism explains approximately 9% of the pigmentation variation observed in this sample. A model-free (unconstricted) analysis indicates that AG heterozygotes decrease skin pigmentation by 1.6 melanin units (p = 0.046) and GG homozygotes by 2.6 melanin units (p = 0.002), with respect to AA homozygotes. The marker rs1800414 remains significant when using the conservative Bonferroni correction (taking into account the intermarker LD patterns and assuming 8 independent tests, the p-value after correction is p = 0.016). Figure 1 shows the distribution of melanin index value by rs1800414 genotype. No association was observed for the other 9 SNPs analyzed in this study.

**Table 3:** Linear regression coefficients and p-values for each of the 10 SNPs in the Canadian East Asian sample.

Gene	Polymorphism	Beta	p-value
*OCA2*	rs7495174 (A/G)	−0.157	0.702
*OCA2*	rs1800414 (A/G)	**−1.256**	**0.002**
*OCA2*	rs1545397 (A/T)	−0.875	0.077
*DCT*	rs1407995 (C/T)	−0.516	0.187
*DCT*	rs2031526 (A/G)	0.557	0.155
*ADAM17*	rs4328603 (C/T)	0.212	0.696
*ADAMTS20*	rs11182091 (C/T)	−0.187	0.625
*ADAMTS20*	rs1510523 (C/T)	0.032	0.942
*ADAMTS20*	rs11182085 (A/G)	−0.235	0.534
*TYRP1*	rs2075509 (A/C)	0.584	0.105

In order to confirm the results of this study, we genotyped the rs1800414 polymorphism in an independent sample of Chinese individuals of Han ancestry recruited in China (N = 207) and measured with a similar instrument. The results of this analysis are reported in Table 4. In agreement with our preliminary results, the linear regression analysis shows that rs1800414 has a significant effect on skin pigmentation, although the effect size is slightly lower than in our study. Under an additive model, each copy of the G allele decreases skin pigmentation by 0.85 melanin units (p = 0.005) and explains around 4% of the variation observed in the sample. Under a model-free (unconstrained) model, the AG heterozygotes decrease skin pigmentation by approximately 1 melanin unit (p = 0.085) and the GG homozygotes by 1.7 melanin units (p = 0.005), with respect to AA homozygotes.

**Table 4:** Observed and expected genotype frequencies, allele frequencies, and Hardy-Weinberg exact test for rs1800414 in the Chinese Han sample.

Gene	Polymorphism	Observed and Expected Genotype Frequencies	Allele Frequencies	Hardy-Weinberg Exact Test (p)	Beta	p-value
		A:A = 51 (42.69)	A = 0.454			
*OCA2*	rs1800414 (A/G)	A:G = 86 (102.63)	G = 0.546	0.024	**−0.855**	**0.005**
		G:G = 70 (61.69)				

We also indicate the regression coefficients and p-values of the linear regression model.

## Discussion

We analyzed the association of 10 SNPs within 5 pigmentation candidate genes (*OCA2*, *DCT*, *ADAM17*, *ADAMTS20* and *TYRP1*) with skin melanin content measured quantitatively in an East Asian sample. Previous studies have indicated that these 5 genes show signatures of natural selection in East Asian populations [16]–[20] and our analysis of signatures of selection using data obtained with the Affymetrix 6.0 chip showed a remarkable agreement with these studies. The 10 SNPs selected for analysis showed high allele frequency differences between East Asian and non-Asian populations and 6 of them (rs1800414, rs7495174, rs1182091, rs1510523, rs11182085 and rs2075509) also had high function, regulatory or phastcons scores in SNPSelector, which indicated that these SNPs could be of functional importance. We observed that one of the markers included in the study, the non-synonymous SNP rs1800414 (His615Arg) located within the *OCA2* gene, was significantly associated with melanin index in our sample of Canadian individuals of East Asian ancestry (p = 0.002). An analysis in an independent sample of Chinese individuals of Han ancestry also showed that the His615Arg polymorphism has a significant effect on skin pigmentation (p = 0.005). Based on both samples, it can be estimated that each copy of the derived G allele (coding for the amino acid Arginine), which is present at high frequency in East Asian populations, but absent in European and West African populations, decreases skin pigmentation by 0.85–1.3 melanin units. Additionally, the unconstrained statistical analysis shows that in terms of its effects on skin pigmentation, this polymorphism fits a codominant model of inheritance, rather than dominant or recessive models. Although significant, the phenotypic effect observed for rs1800414 is lower than the effect that has been reported for other polymorphisms previously associated with skin pigmentation. For example, studies in African American populations have shown that polymorphisms located within the pigmentation genes SLC24A5, SLC45A2 and KITLG have an effect of more than 3 melanin units per allele copy [3],[5],[6]. However, direct comparison between studies is complicated by the different pigmentation characteristics of the samples. This is one of the first formal reports of association with skin pigmentation measured using reflectometry in East Asian populations. Our study indicates that the *OCA2* gene was *independently* involved in the evolution of light pigmentation in Europe and East Asia, and in combination with previous findings for other genes (*SLC24A5* and *SLC45A2*), strongly suggests that there was convergent evolution towards light pigmentation in Europe and East Asia. Previous studies reported that the *OCA2/HERC2* gene showed distinct signatures of positive selection in Europe and East Asia [16],[19],[20],[21]. Markers in the *HERC2* gene are associated with blue eyes in European and related populations. In particular, the SNP rs12913832 segregates almost perfectly with blue-brown eye color [24]–[26]. This SNP is located within a highly conserved region that may act as a control region for *OCA2* and a recent study reported that rs12913832 had a significant effect on the levels of *OCA2* mRNA [25],[27]. Lao et al. [19] reported that the *OCA2* gene had significant Extended Haplotype Homozygosity (EHH) values in European and East Asian samples, but the core haplotypes were different in both populations. Yuasa et al. [28] noted that the rs1800414 G allele (R615) is very frequent in East Asian populations, but rare or absent in African and Indo-European populations. Anno et al. [29] also showed that European and East Asian populations are characterized by different haplotypes at the *OCA2* gene, with the East Asian haplotype harboring the variant rs1800414 G, which is the allele that is associated with light skin in our study. More recently, Donelly et al. [21] described that the rs1800414 allele is under selection in East Asia, and the blue eye allele BEH2 (defined by rs12913832) is under selection in Europe and Southwest Asia. Therefore, it seems clear that there were independent selective processes acting on the *OCA2* gene in Europe and East Asia, involving distinct haplotypes.

Our sample comprises individuals of East Asian ancestry living in Toronto. The majority of the subjects have ancestry from China, South Korea and Japan (N = 96), but some individuals have ancestry from Southeast Asia (Vietnam, Thailand and Phillipines, N = 26). If there are large differences in frequency between East Asian populations for rs1800414, our significant association results for this SNP could be confounded by population stratification. However, two observations indicate that this is not the case: 1/ There are no significant deviations from Hardy-Weinberg proportions for any of the markers included in our study (Table 2). The effect of allele frequency differences between East Asian populations would have been reflected in deviations from Hardy-Weinberg (excess of homozygotes, Wahlund effect). In fact, there is a slight excess of heterozygotes for rs1800414 in our total sample, which is the opposite of what would be expected in the presence of stratification, 2/ The statistical analysis excluding the Southeast Asian subjects (N = 96) is also significant and shows remarkable concordance with the results obtained using the full sample (beta = −1.6, p = 0.001). In this respect, it is important to note that Yuasa [28] reported that there are no large frequency differences between five samples from China and Japan for the rs1800414 G allele (44.8%–63%). Similarly, we did not observe significant allele frequency differences between the Canadian East Asian sample and the Chinese sample that was used for replication (p = 0.255).

Our statistical analysis was significant for rs1800414, but not for the other SNPs genotyped in this sample, including 2 additional SNPs within the *OCA2* gene. Given our relatively small sample size, our study was not adequately powered to identify loci with small effects. The rs1800414 polymorphism explains a substantial proportion of the skin pigmentation variation observed in the sample. A relevant question is if the observed effects are due to rs1800414 or to a causative SNP in LD with rs1800414 within the *OCA2* gene. In this sense, there is strong evidence pointing to rs1800414 as the causative variant itself. In addition to SNPSelector, we used other tools to infer the functional effect of this polymorphism (FastSNP, the SNP function portal, SIFT and Polyphen). All of these methods suggest that this non-synonymous rs1800414 SNP, which was first described by Lee et al. [30], is functionally important. FastSNP indicates that the functional effect of this SNP may be mediated through the regulation of alternative splicing. The programs Polyphen and SIFT also point to a damaging effect of the A to G transition at rs1800414. It would be extremely important to carry out gene expression studies of this polymorphism, similar to the research published for other variants known to be associated with skin pigmentation using primary cultures of human melanocytes [2],[27],[31].

Our study provides new evidence regarding the genetic and evolutionary processes driving the lightening of skin following the migration of anatomically modern humans from Africa to high latitude regions in Europe and East Asia. Evidence is growing that the reduction in melanin content took place, at least in part, independently in these two regions. We now know that the evolution of skin pigmentation has been quite complex: some genes were the target of positive selection only in one population group (eg. *SLC24A5* and *SLC45A2* in Europe), whereas other genes were under selection independently in more than one group (eg. *OCA2* in Europe and East Asia). However, there are still many aspects of the evolution of skin pigmentation that remain unclear. Our picture of the genetics of normal pigmentation variation in non-European populations is still incomplete, and the evolutionary time frame remains to be elucidated. When did the evolution to light skin take place in Europe and East Asia? It has been suggested, based on evidence collected for the *SLC24A5* gene, that the evolution to light skin occurred in Europe long after the arrival of anatomically modern humans to this continent [32], but it will be necessary to collect information on additional genes and from different geographic regions to gain a better understanding of the evolution of skin pigmentation in human populations.

## Materials and Methods

### Ethics statement

Written informed consent was obtained from each participant, and the study was approved by the University of Toronto Health Sciences Research Ethics Board.

### Recruitment

Participants were recruited by the Molecular Anthropology Laboratory at the University of Toronto Mississauga (UTM) between 2007 and 2009. Recruitment took place primarily through the use of advertisements on UTM campus, and online advertisements in the University of Toronto community. Geographic origin was assessed using questions regarding the participant's place of birth and the ancestry of their parents and maternal and paternal grandparents. In total, 122 East Asians were recruited.

### Measurement of melanin using reflectometry

We took quantitative melanin measurements from each participant's inner arm using a narrow-band reflectometer (DermaSpectrometer, Cortex Technology, Hadsund, Denmark). This instrument emits light at the green (568 nm) and red (655 nm) wavelengths of the visible spectrum and a photodetector measures the amount of light reflected by the skin. These measurements are used to estimate the melanin content in the skin, which is expressed as the Melanin Index (M). In human populations, the melanin index ranges from the low 20s (individuals with light skin) to close to 100 (individuals with dark skin). Throughout the text, when we refer to melanin units, we refer to the melanin index values obtained with the DermaSpectrometer. More information about this instrument is available in Shriver and Parra [33]. In order to capture the most accurate reading of constitutive skin pigmentation, these measurements were carried out during the winter.

### Selection of SNPs in pigmentation candidate genes

We used SNPSelector [http://snpselector.duhs.duke.edu/hqsnp36.html] to prioritize a limited number of SNPs to genotype within each of the pigmentation candidate genes. SNPSelector is a SNP selection tool that provides information on population allele frequencies, linkage disequilibrium patterns, potential SNP function and patterns of SNP conservation. Our criteria for SNP selection was based on: 1/ high frequency differences between East Asian and non-Asian populations (West Africa and Europe) and 2/ potential functional effect, based on the function score, regulatory score or conservation score (PhastCons score). The following SNPs were selected for genotyping: 1/ Gene *OCA2*: rs1800414 is a non-synonymous polymorphism with an allele present at high frequency in East Asian populations (G allele, 59%) but absent in non-Asian populations. This SNP also had very high function, regulatory and phastcons scores; rs1545397 is an intronic polymorphism showing dramatic allele frequency differences between East Asian and non-Asian populations (>85%); rs7495174 is an intronic variant showing high frequency differences between East Asian and non-Asian populations (>45%) and a high regulatory score (CpG island), 2/ Gene *DCT*: rs1407995 and rs2031526 are intronic polymorphisms showing very high frequency differences between East Asian and non-Asian populations (>60%), 3/Gene *ADAM17*: rs4328603 is an intronic SNP showing very high frequency differences between East Asian and non-Asian populations (>60%), 4/ Gene *ADAMTS20*: rs11182091 is an intronic SNP with substantial frequency differences between East Asian and non-Asian populations (>30%) and a high regulatory score (conserved transcription factor binding site); rs1510523 is an intronic variant with high frequency differences between East Asian and non-Asian populations (>40%) and high regulatory and phastcons scores; rs11182085 is an intronic SNP with substantial frequency differences between East Asian and non-Asian populations (>30%) and high regulatory and phastcons scores, 5/ Gene *TYRP1*: rs2075509 is an intronic variant with high frequency differences between East Asian and non-Asian populations and high regulatory and phastcons scores. No markers were studied at the *KITLG* gene because no SNPs were identified with large frequency differences between East Asian and European populations. This is consistent with reports indicating that the signatures of selection observed in *KITLG* region are shared in Europeans and East Asians [6].

### DNA collection and genotyping

A sample of each participant's blood was collected in a 4-mL EDTA tube. DNA was extracted from the blood using the E.Z.N.A. Blood DNA Midi Kit (Omega Bio-Tek, Georgia, United States). Genotyping was done by the company KBiosciences [http://www.kbioscience.co.uk/] using a KASPar assay that relies on competitive allele specific PCR and fluorescent detection. Eighty-nine genotypes were characterized in duplicate, and the concordance rate between the samples and the blind duplicates was 100%.

### Statistical analysis

Departures from Hardy-Weinberg proportions were evaluated using an exact test available at the website http://ihg2.helmholtz-muenchen.de/cgi-bin/hw/hwa1.pl. Linkage disequilibrium (LD) between the markers located within the same genes was estimated using the program EMLD (University of Texas, Houston, TX). LD is reported as the r^2^ value. Association between the selected SNPs and Melanin Index was tested using linear regression. Sex was included as a covariate, as it has been found to be associated with skin pigmentation in previous studies [34],[35]. Each of the 10 SNPs was tested independently using additive and unconstrained models. The regression analysis was carried out with the program SPSS (version 17.0, SPSS Inc., 2008).

### Power analysis

We used the program Quanto [http://hydra.usc.edu/gxe/] to estimate the statistical Power of our study using an additive model and a range of allele frequencies and allelic effects (measured as the regression coefficient-beta). These estimates are based on the distribution of melanin levels observed in the East Asian sample (mean melanin index = 31, standard deviation = 3) and a sample size of 120 individuals. For markers with intermediate allele frequencies (35%–65%), our study has more than 90% power to detect effects higher than 1.3 melanin units (type I error rate = 0.05, two-sided test). The Power drops for markers with more extreme frequencies: for a marker with 20% frequency, the power to identify effects higher than 1.3 is 77.8% and for a marker with 10% frequency, it is 52.5%.

### Replication of significant results

For replication of the significant results of the initial analysis, the OCA2 His615Arg polymorphism was genotyped by sequencing in an independent sample from China. The sample comprised 207 individuals of Han ancestry that were recruited by Professor Li Jin at Fudan University. Skin pigmentation was measured in the inner upper arm with an instrument similar to that used to measure pigmentation in the Canadian East Asian samples (DermaSpectrometer, Cortex Technology, Hadsund, Denmark). Informed consent was obtained from each participant, and the project approved by the research ethics board of the School of Life Sciences, Fudan University.

### Tests of positive selection

Four different tests of selection were used to evaluate evidence of positive selection in the HapMap East Asian sample for the five genes analyzed in this study. They include the locus-specific branch length (LSBL), the log of the ratio of heterozygosities (ln*RH*), Tajima's *D*, and whole genome long range haplotype (WGLRH) test [36]–[39]. The results reported here are based on genome-wide data for the East Asian, European and West African HapMap samples obtained with the Affymetrix 6.0 chip, which includes approximately 1 million SNPs. The LSBL test evaluates if genetic markers within a genomic region show unusual levels of differentiation with respect to the genome average. This test apportions the genetic variation observed in East Asian, European and West African populations for each SNP, and identifies markers with high levels of genetic differentiation in the East Asian sample. The ln*RH* test highlights genomic regions with low levels of genetic diversity in the population of interest, in comparison with other population groups. This statistic was calculated for a two-way population comparison between East Asians and Europeans, and East Asians and West Africans, using an overlapping sliding window size of 100,000 base pairs (bp) and moving in 25,000 bp increments along a chromosome. Regions of the genome with negative Tajima's *D* values are also a hallmark of positive selection. However, negative values of *D* can result from demographic events as well, specifically the recovery from a population bottleneck. For this reason, it is important to compare local values of Tajima's *D* with the empirical levels observed in the genome. As for the ln*RH* analysis, Tajima's *D* was calculated for each population using an overlapping sliding window size of 100,000 bp with a 25,000 bp offset. The statistical significance for each of the LSBL, ln*RH*, and Tajima's *D* statistics was based on the genome-wide empirical distribution, using the formula PE (x) = (number of loci>x)/(total number loci). The final test used to infer selection is the WGLRH test of Zhang et al. [37]. This test first calculates the Relative Extended Haplotype Homozygosity (REHH) for each core haplotype in the data set and identifies core haplotypes with longer than expected ranges of linkage disequilibrium (LD) given their frequency in the population. A gamma distribution is then estimated using maximum likelihood methods against which the REHH of each core haplotype is tested to determine if its respective p-value is suggestive of recent, positive selection. This test then considers the ancestral state of the alleles, determined by a closely related outgroup, to identify SNPs where the derived allele has risen to high frequencies (>0.60). For this data set, the ancestral state for all SNPs available in the chimpanzee sequence was retrieved using the UCSC genome browser. In total, the ancestral states for 846,032 SNPS on the autosomes and X chromosome were obtained. Lastly, the WGLRH test applies a false discovery rate approach to control for false positives and identifies significant extended haplotypes. The four statistics used in this analysis have been described in more detail in Bigham et al. [40].

### Prediction of potential functional effects

We analyzed the OCA2 His615Arg polymorphism using the programs FastSNP (http://fastsnp.ibms.sinica.edu.tw/pages/input_CandidateGeneSearch.jsp), the SNP function portal (http://brainarray.mbni.med.umich.edu/Brainarray/Database/SearchSNP/snpfunc.aspx) and SIFT (http://sift.jcvi.org/) in order to predict potential functional effects.

## References

[1] Parra EJ (2007). Human pigmentation variation: evolution, genetic basis, and implications for public health.. Am J Phys Anthropol Suppl.

[2] Sturm RA (2009). Molecular genetics of human pigmentation diversity.. Hum Mol Genet.

[3] Lamason RL, Mohideen MA, Mest JR, Wong AC, Norton HL (2005). SLC24A5, a putative cation exchanger, affects pigmentation in zebrafish and humans.. Science.

[4] Graf J, Hodgson R, van Daal A (2005). Single nucleotide polymorphisms in the MATP gene are associated with normal human pigmentation variation.. Hum Mutation.

[5] Norton HL, Kittles RA, Parra E, McKeigue P, Mao X (2007). Genetic evidence for the convergent evolution of light skin in Europeans and East Asians.. Mol Biol Evol.

[6] Miller CT, Beleza S, Pollen AA, Schluter D, Kittles RA (2007). Cis-regulatory changes in Kit Ligand expression and parallel evolution of pigmentation in sticklebacks and humans.. Cell.

[7] Shriver MD, Parra EJ, Dios S, Bonilla C, Norton H (2003). Skin pigmentation, biogeographical ancestry and admixture mapping.. Hum Genet.

[8] Akey JM, Wang H, Xiong M, Wu H, Liu W (2001). Interaction between the melanocortin-1 receptor and P genes contributes to inter-individual variation in skin pigmentation phenotypes in a Tibetan population.. Hum Genet.

[9] Makova K, Norton H (2005). Worldwide polymorphism at the MC1R and normal pigmentation variation in humans.. Peptides.

[10] Kanetsky PA, Swoyer J, Panossian S, Holmes R, Guerry D (2002). A polymorphism in the agouti signaling protein gene is associated with human pigmentation.. Am J Hum Genet.

[11] Bonilla C, Boxill LA, Donald SA, Williams T, Sylvester N (2005). The 8818G allele of the agouti signaling protein (ASIP) gene is ancestral and is associated with darker skin color in African Americans.. Hum Genet.

[12] Voisey J, Gomez-Cabrera M del C, Smit DJ, Leonard JH, Sturm RA (2006). A polymorphism in the agouti signaling protein (ASIP) is associated with decreased levels of mRNA.. Pigment Cell Res.

[13] Han J, Kraft P, Nan H, Guo Q, Chen C (2008). A genome-wide association study identifies novel alleles associated with hair color and skin pigmentation.. PLoS Genet.

[14] Stokowski RP, Pant PV, Dadd T, Fereday A, Hinds DA (2007). A genomewide association study of skin pigmentation in a South Asian populations.. Am J Hum Genet.

[15] Voight BF, Kudaravalli S, Wen X, Pritchard JK (2006). A map of recent positive selection in the human genome.. PLoS Biol.

[16] McEvoy B, Beleza S, Shriver MD (2006). The genetic architecture of normal variation in human pigmentation: an evolutionary perspective and model.. Hum Mol Genet.

[17] Izagirre N, Garcia I, Junquera C, de la Rua C, Alonso S (2006). A scan for signatures of positive selection in candidate loci for skin pigmentation in humans.. Mol Biol Evol.

[18] Myles S, Somel M, Tang K, Kelso J, Stoneking M (2007). Identifying genes underlying pigmentation differences among human populations.. Hum Genet.

[19] Lao O, de Gruijter JM, van Duijn K, Navarro A, Kayser M (2007). Signatures of positive selection in genes associated with human skin pigmentation as revealed from analyses of single nucleotide polymorphisms.. Ann Hum Genet.

[20] Alonso S, Izagirre N, Smith-Zubiaga I, Gardeazabal J, Diaz-Ramon JL (2008). Complex signatures of selection for the melanogenic loci TYR, TYRP1 and DCT in humans.. BMC Evol Biol.

[21] Donnelly MP, Speed WC, Kidd JR, Pakstis AJ, Kidd KK (2009). Selection for blue eyes in Europe and light skin pigmentation in East Asia at OCA2/HERC2. Platform Abstract #294.. http://www.ashg.org/2009meeting/pdf/platforms_4up.pdf.

[22] Kelley JL, Madeoy J, Calhoun JC, Swanson W, Akey JM (2006). Genomic signatures of positive selection in humans and the limits of outlier approaches.. Genome Res.

[23] Teshima KM, Coop G, Przeworski M (2006). How reliable are empirical genomic scans for selective sweeps?. Genome Res.

[24] Eiberg H, Troelsen J, Nielsen M, Mikkelsen A, Mengel-From J (2008). Blue eye color in humans may be caused by a perfectly associated founder mutation in a regulatory element located within the HERC2 gene inhibiting OCA2 expression.. Hum Genet.

[25] Sturm RA, Duffy DL, Zhao ZZ, Leite FP, Stark MS (2008). A single SNP in an evolutionary conserved region within intron 86 of the HERC2 gene determines human blue-brown eye color.. Am J Hum Genet.

[26] Kayser M, Liu F, Janssens AC, Rivadeneira F, Lao O (2008). Three genome-wide association studies and a linkage analysis identify HERC2 as a human iris color gene.. Am J Hum Genet.

[27] Cook AL, Chen W, Thurber AE, Smit DJ, Smith AG (2009). Analysis of cultured human melanocytes based on polymorphisms within the SLC45A2/MATP, SLC24A5/NCKX5, and OCA2/P loci.. J Invest Dermatol.

[28] Yuasa I, Umetsu K, Harihara S, Kido A, Miyoshi A (2007). Distribution of two Asian-related coding SNPs in the MC1R and OCA2 genes.. Biochem Genet.

[29] Anno S, Abe T, Yamamoto T (2008). Interactions between SNP alleles at multiple loci contribute to skin color differences between Caucasoid and Mongoloid subjects.. Int J Biol Sci.

[30] Lee ST, Nicholls RD, Jong MT, Fukai K, Spritz RA (1995). Organization and sequence of the human P gene and identification of a new family of transport proteins.. Genomics.

[31] Sviderskaya EV, Bennett DC, Ho L, Bailin T, Lee ST, Spritz RA (1997). Complementation of hypopigmentation in p-mutant (pink-eyed dilution) mouse melanocytes by normal human P cDNA, and defective complementation by OCA2 mutant sequences.. J Invest Dermatol.

[32] Gibbons A (2007). American Association of Physical Anthropologists meeting. European skin turned pale only recently, gene suggests.. Science.

[33] Shriver MD, Parra EJ (2000). Comparison of narrow-band reflectance spectroscopy and tristimulus colorimetry for measurements of skin and hair color in persons of different biological ancestry.. Am J Phys Anthropol.

[34] Jablonski NG, Chaplin G (2000). The evolution of human skin coloration.. J Hum Evol.

[35] Roh K, Kim D, Ha S, Ro Y, Kim J, Lee H (2001). Pigmentation in Koreans: study of the differences from caucasians in age, gender and seasonal variations.. Br J Dermatol.

[36] Tajima F (1989). Statistical method for testing the neutral mutation hypothesis by DNA polymorphism.. Genetics.

[37] Zhang C, Bailey DK, Awad T, Liu G, Xing G (2006). A whole genome long-range haplotype (WGLRH) test for detecting imprints of positive selection in human populations.. Bioinformatics.

[38] Shriver M, Kennedy GC, Parra EJ, Lawson HA, Huang J (2004). The genomic distribution of population substructure in four populations using 8,525 autosomal SNPs.. Human Genomics.

[39] Storz JF, Payseur BA, Nachman MW (2004). Genome scans of DNA variability in humans reveal evidence for selective sweeps outside of Africa.. Mol Biol Evol.

[40] Bigham A, Mao X, Brutsaert T, Wilson M, Julian CG (2009). Identifying positive selection candidate loci for high-altitude adaptation in Andean populations.. Human Genomics.

